# The magnitude of opportunistic infections and associated factors in HIV-infected adults on antiretroviral therapy in southern zone Tigray, Ethiopia: a cross-sectional study

**DOI:** 10.11604/pamj.2020.35.126.17839

**Published:** 2020-04-17

**Authors:** Teklay Zeru Weldearegawi, Hadgu Gerensea, Hagos Berihu, Gebreamlak Gidey, Mebrahtom Zeru Welearegay

**Affiliations:** 1 Department of Nursing, College of Health Sciences, Aksum University, Aksum, Ethiopia; 2 Department of Midwifery, College of Health Sciences, Aksum University, Aksum, Ethiopia; 3 Department of Biomedical Science, College of Health Sciences, Adigrat University, Adigrat, Ethiopia

**Keywords:** AIDS, opportunistic infections, WHO clinical stage, CD4 count, southern zone Tigray

## Abstract

**Introduction:**

Greater than twenty known opportunistic infections have been associated with HIV infection and usually patients experience co-infections during the stage of illness, HIV-related opportunistic infections are associated with significant morbidity and mortality.

**Methods:**

A hospital-based retrospective study was conducted in HIV-infected adult patients on antiretroviral (ART) from April to June 2017; secondary data were collected from review of clinical records. A total of 400 study participants selected through a systematic sampling technique and a pre-tested checklist was used to collect data from records of study subjects. The data was entered and analyzed using SPSS version 22.

**Results:**

A total of 400 patients included in the study, in which more than half (51.0%) were females. The mean age of patients was 34 (standard deviation [SD] ±1.96) years. The overall of opportunistic infections (OIs) among HIV/AIDS patients on ART was 55.3%. The highest rates of OIs observed were oral candidacies 11.0%, followed by herpes zoster (10.8%) and tuberculosis (TB) (9.5%). The odds of having college and above educational levels were less likely to developed OIs compared to illiterate (AOR=0.007; 95% CI=0.053, 0.634). The odds of having OIs in WHO clinical stage I were less likely to have OIs compared to WHO clinical stage III and V (AOR=0.001; 95% CI=0.000, 0.015 and AOR=0.00; 95%CI=0.00 respectively).

**Conclusion:**

There was a high prevalence of OIs observed in this study. Illiterate educational level and advanced WHO clinical stages were found to be predictors of OIs. Interventions were aimed at promoting early HIV testing and ART enrollment of HIV-infected.

## Introduction

The clinical symptoms of HIV infection encompass a spectrum ranging from primary infection and prolonged non-symptomatic state to advanced disease [1,2]. Most of the opportunistic infections (OIs), usually manifest at a terminal stage of the illness, are the commonest causes of morbidity and mortality among HIV/AIDS infected patients. Despite the existence of prevention and treatment modalities, opportunistic infections continued to be the leading causes of morbidity and mortality among people living with HIV/AIDS [1,3]. The risk for the development of OIs in HIV patients depends on exposure to potential pathogens, the virulence of the pathogens, the degree of host immunity, and the use of antimicrobial prophylaxis [4]. HIV causes progressive depletion of the CD4 T cells, which leads to life-threatening opportunistic infections (OIs) or malignancies during the natural course of the disease greater than 90% of them are responsible for the development of AIDS morbidities and mortalities [5].

The World Health Organization (WHO) reports the number of people living with HIV/AIDS has increased from 33.3 million in 2010 to 36.7 million in 2015 including 2.1 million newly infected people. Developing countries share 69% of the global HIV burden [5]. Greater than 20 specific opportunistic infections have been associated with HIV infection and usually, patients experience co-infections during their illness [6,7]. Stage II infections such as herpes zoster (HZ) and other skin infections occur in early World Health Organization (WHO) clinical stages (stages I and II) [2,7,8] whereas critical, life-threatening infections such as CNS toxoplasmosis and cryptococcal meningitis come in later WHO clinical stages (stages III and IV) with severe immunity suppression. Some life-threatening infections, such as pneumonia and tuberculosis (TB), may occur in early as well as in later WHO clinical stages [9-12]. The most common opportunistic diseases in HIV patients in Ethiopia are tuberculosis (TB), oral candidacies, Pneumocystis Carini Pneumonia (PCP), bacterial pneumonia, kaposi´s sarcoma and lymphoma [13,14].

HIV/AIDS-related opportunistic infections are the predominant causes of morbidity and mortality and virtually cannot be a cure, except life-long suppressive therapy after an acute infection. Prevention of such illness through primary prophylaxis is, therefore, compelling [15]. Although nationally representative and comprehensive data regarding the magnitude of opportunistic infections lack in Ethiopia, the same regional studies have shown the prevalence ranging from 19.7% to 48% [15-17]. Antiretroviral therapy (ART) increases the length and quality of life and work productivity of patients by improving survival and decreases the incidence of OIs in HIV-infected people through reduction of the viral load and increasing the level of CD4 cells [18]. The widespread use of ART has had the most profound influence on reducing OI-related mortality in HIV-infected persons in those countries in which these therapies are accessible and affordable. Worldwide, it is estimated that between 250,000 and 350,000 deaths were averted in 2005 as a result of increased treatment access, however, OIs continued to cause morbidity and mortality in HIV/AIDS patients even after receiving ART.

This mortality occurs because some patients do not have a sustained response to antiretroviral agents for multiple reasons including poor adherence, drug toxicities, drug interactions or initial acquisition of a drug-resistant strain of HIV-1 [7,19]. The adult HIV prevalence of Ethiopia was estimated to be 1.1% in 2015, where more than 691 thousand people are near to be suffering from the disease. It is the second leading cause of death in Ethiopia resulting in the death of greater than 26 thousand people in 2015. More than 90% of HIV/AIDS deaths are contributed to opportunistic infections and malignancies [20]. The type of opportunistic infection which affects people living with HIV/AIDS different from region to region [21]. Therefore, for the strategies in HIV/AIDS-related morbidity and mortality to be decreased, identifying of opportunistic infections, their frequency and distribution play a significant role. Therefore, this research assessed the prevalence of opportunistic infections among people living with HIV/AIDS so that responsible governmental and nongovernmental stakeholders and communities who have similar setting can use the result of this research for further planning and implementation of control HIV/AIDS and related death.

## Methods

The study area, design and period south zone Tigray is located in the northern part of Ethiopia, which is 821 kilometers away from the capital city Addis Ababa. It had a projected total population of people in the year 2016. There are four hospitals and ten health centers in the zone (Tigray regional health bureau state health bureau, unpublished data, 2016). The health service coverage of the region is estimated to be about 100%. An ART program was launched on March 26, 2006, in the region. Lemlem Karl Hospital, Korem Hospital, Alamata, which are the facilities in the southern zone providing ART services to HIV/AIDs patients, began providing these services in March 2006. Until June 2017, a total of 6109 HIV/AIDS patients have utilized ART services. A hospital-based, retrospective study was conducted in the southern zone hospital ART clinic from April to June 2017. A total of 400 study participants were selected by systematic random sampling using the ART registration book as a sampling frame, and these patients´ clinical records were reviewed. Data were collected using a checklist, which was adopted from the hospital´s clinical record format for monitoring HIV/AIDS patients on ART.

Information on patients´ details, such as socio-demographic characteristics, functional status and type of OIs, prophylaxis usage and baseline WHO clinical staging, CD4 cell count, hemoglobin level and weight were retrieved from clinical records of the HIV/AIDS patients by trained nurses. Those patients´ clinical records that were not complete or were missing, data were omitted and were replaced with the next patient´s record on the list. The data collection format was checked for its completeness and consistency with the patient´s clinical records by a supervisor and the investigators daily. Data were double entered into a data entry file using SPSS software version 22 and were analyzed according to the different variables. Results were presented by using mean, standard deviation (SD) and simple frequency les with percentages. The prevalence of OIs was determined as the proportion of HIV/AIDS patients on ART who developed one or more OIs. Univariate and multivariate analysis logistic regression models were used to describe the significance of the association between the prevalence of OIs between selected variables. Crude and adjusted odds ratios (CORs and AORs, respectively) with 95% confidence intervals (CIs) were used to describe the strength of association between the selected study variables. The criterion for significance was set at P, 0.05 based on a two-sided test.

## Results

**Socio-demographic and clinical characteristics of the study participants:** a total of four hundred HIV/AIDS patients´ ART records were reviewed in the current study. The mean age of study participants was 35.5 (SD±6.8) years and ranging from 18-71 years. Most of the patients were in the age group of 18-29 years (44.0%), were female (51.0%), WHO clinical stage I, (54.5%), urban (87.0%) and had an elementary school education (38.0%). Many study participants (150, or 41.9%) were at WHO clinical stage III. Concerning functional status, most (79.3%) of patients were working for personnel. Regards to marital status 52.5% of participants were single. The majority (94.8%) of the participants were receiving Cotrimoxazole, while 5.3% were receiving Isonicotinylhydrazine (INH) prophylaxis. About seventy-two percent of participants had a CD4 count of 200 cells/mm^3^. In addition, about 88.3% of participants had, 10 mg/dL hemoglobin level and 55.3% weighed under 60 kg (Table 1).

**Table 1:** Socio-demographic and clinical characteristics of the study participants (n=400) among HIV/AIDS patients on ART clinic at general hospital southern zone Tigray, Northern Ethiopia, 2017

Variables	Category	Number	Percent (%)
Sex	Male	196	49.0
	Female	204	51.0
Marital status	Single	210	52.5
	Married	138	34.5
	Divorced	29	7.3
	Widowed	23	5.8
Educational status	Illiterate	85	21.3
	Primary school (1^st^–8^th^ grade)	152	38.0
	Secondary school (9^th^–12^th^ grade)	109	27.3
	College/University	54	13.5
Occupation	No work	63	15.8
	Daily labor	117	29.0
	Private business	95	23.8
	Government employee	126	31.5
Residence	Urban	348	87.0
	Rural	52	13.0
Age group, years	18–29	176	44.0
	30–39	110	27.5
	40–49	80	20.0
	$50	34	8.5
WHO stage	I	218	54.5
	II	82	20.3
	III	82	20.5
	IV	19	4.8
Functional status	Working	317	79.3
	Ambulatory	62	15.5
	Bedridden	21	5.3
OI prophylaxis	Cotrimoxazole	379	94.8
	INH	21	5.3
Baseline CD4 cell count, cells/mm^3^*	<200	109	27.3
	$200	291	72.8
Baseline hemoglobin level, g/dL*	<10	47	11.8
	$10	353	88.3
Baseline weight, kg	<60	44.8	55.3
	$60	179	44.7

Note: *Percentage was not calculated for total study participants (400), due to missing values

**Prevalence of OIs:** out of four hundred patients, 195 had diagnosed OIs, with an overall prevalence of 48.75% (195/400). There were a total of 221 OIs diagnosed in the 195 patients. About 41.5% (166/400), 13.75% (55/400) of the study participants had single and multiple OIs, respectively. The most frequent OIs were oral candidacies and ulcers mouth, genital (11%) (44/400) followed by herpes zoster 10.8% (43/400) and tuberculosis at 9.5% (38/400) (Table 2).

**Table 2:** Prevalence of opportunistic infections among HIV/AIDS patients on ART clinic, at general hospitals of the southern zone of Tigray, Ethiopia, 2017

Opportunistic infections	Number	Percent (%)
Herpes zoster	43	10.8
Tuberculosis	38	9.5
Bacterial pneumonia	28	7.0
Oral candidacies	44	11.0
Chronic diarrhea	1	1.8
Pneumocystis carini pneumonia	11	2.8
CNS toxoplasmosis	12	3.0
Cryptococcal meningitis	7	1.8
Tuberculosis, herpes zoster, and chronic diarrhea	11	2.8
Upper respiratory tract infection	23	5.8
Kaposi sarcoma	3	0.8

**Risk factors of OIs:** the prevalence of OIs among males (54.0%) and females (56.4%) was comparable. The highest prevalence of OIs was found in those individuals divorced (79.3%), the age group of 18.29 and 30-39 years old (56.8%) (57.3%) respectively more or less similar, with secondary school educational level (57.4%), rural dwellers (57.7%) and with bodyweight >60kg (59.0%). However, the prevalence of OIs was not statistically different among the above variables (P.0.05). In bivariate analysis, study participants in marital status, divorced were more likely to develop OIs than single and married (COR=0.256; 95% CI=0.100, 0.654), those secondary school, college and above were less likely to develop OIs at (COR=0.206; 95% CI=0.112, 0.379) and COR=0.181; 95% CI=0.085, 0.386, respectively). Concerning WHO clinical stages III and IV were more likely to develop OIs compared to those participants at WHO clinical stages I (COR=0.003; 95% CI=0.00, 0.021 and COR=0.00; 95% CI=0.00; 0.00, 0.00, respectively). Those participants with CD4 cell count of <200 cells/mm^3^ were more likely to develop OIs than their counterparts with higher CD4 cell count (COR=1.347; 95% CI=0.861, 2.108). Participants who were 50 and above years old age (COR=1.667; 95% CI=0.795, 3.492) were more likely to develop OIs compared to patients 18-29 years old. In addition, participants who were >60kg weight were less likely develop OIs (COR=0.640; 95% CI=0.426, 0.963) compared to their counterparts who were <60kg (Table 3).

**Table 3:** Bivariate analysis factors associated with opportunistic infections (OIs) among HIV/AIDS patients taking antiretroviral therapy (ART) at general hospitals southern zone Tigray, Northern Ethiopia, 2017

Variable	Category	OI		COR (95% CI)	P-value
		Yes	No		
Sex	Male	106(54.0%)	90(45.9%)	1.097(.0749, 1.627)	0.645
	Female	115(56.4%)	89(43.6%)	1	
Marital status	Single	104(49.5%)	106(50.5%)	1	
	Married	78(56.5%)	60(43.5%)	0.755(0.490, 1.162)	0.004
	Divorced	23(79.3%)	6(20.7%)	0.256(0.100, 0.654)	
	Widowed	16(69.6%)	7(30.4%)	0.429(0.429, 1.086)	
Occupation	No work	34(54.0%)	29(46.0%)	1	
	Daily labor	58(50.0%)	58(50.0%)	1.172(0.634, 2.168)	0.45
	Private business	57(60%)	38(40.0%)	0.782(0.441, 1.487)	
	Government employee	72(57.1%)	54(42.9%)	0.879(0.479, 1.615)	
Educational status	Illiterate	29(34.1%)	56(65.9%)	1	.00
	Primary	74(48.7%)	78(51.3%)	0.546(0.315, 0.946)	
	Secondary	78(71.6%)	31(28.4%)	0.206(0.112, 0.379)	
	College and above	40(25.9%)	14(25.9%)	0.181(0.085, 0.386)	
Residence	Urban	191(54.9%)	157(45.1%)	1	
	Rural	30(57.7%)	22(42.3%)	0.892(0.495, 1.608)	0.704
WHO stage	I	40(18.3%)	178(81.7%)		
	II	81(100%)	0(00%)	1	0.000
	III	81(98.8%)	1(1.2%)	0.00	
	IV	19(100%)	0(00%)	0.003(0.00, 0.021)	
OI prophylaxis was given**	Yes	206(54.4%)	173(45.9%)	1	
	No	15(71.4%)	6(28.6%)	0.476(0.18, 1.254)	0.133
Baseline CD4 cell, cells/mm^3^	<200	66(60.6%)	43(39.4%)	1	
	$200	155(53.3%)	136(46.7%)	1.347(0.861, 2.108)	0.193
Functional status	Working	170(54.3%)	143(45.7%)	1	
	Ambulatory	36(60.0%)	24(40.0%)	0.793(0.453, 1.391)	0.418
	Bedridden	15(55.6%)	12(44.4%)	0.951(0.431, 2.099)	
Age (years)	18-29	100(56.8%)	76(43.21%)	1	
	30-39	63(57.3%)	47(42.7%)	0.982(0.607, 1.588)	0.176
	40-49	43(53.8%)	37(46.3%)	1.132(0.666, 1.926)	
	$50	15(44.1%)	19(55.9%)	1.667(0.795, 3.492)	
Baseline HGB level, g/dL	<10	21(44.7%)	26(55.3%)	1	
	$10	200(56.7%)	153(43.3%)	0.618(0.335, 1.140)	0.123
Baseline weight, kg	<60	72(48.3%)	77(51.7%)	1	
	$60	149(59.4%)	102(40.6%)	0.640(0.426, 0.963)	0.032

Note:**OI prophylaxis included Cotrimoxazole and/or INH

**Associated factors of OIs:** multivariable analysis showed that the WHO clinical stage was significantly associated with the presence of OIs (P<0.001). The odds of college and above educational level were less likely to develop OIs compared to illiterate (AOR=0.007; 95% CI=0.053, 0.634). The odds of having OIs in WHO clinical stage I were less likely to have OIs compared to WHO clinical stage III and V (AOR=0.001; 95% CI=0.000, 0.015 and AOR=0.00; 95%CI=0.00 respectively) (Table 4).

**Table 4:** Multivariate analysis factors associated with opportunistic infections (OIs) among HIV/AIDS patients taking antiretroviral therapy (ART) at general hospitals southern zone Tigray, Ethiopia, 2017

Variables	Category	OI		AOR (95% CI)	P-value
		Yes	No		
Marital status	Single	104(49.5%)	106(50.5%)	1	
	Married	78(56.5%)	60(43.5%)	0.5879(0.254, 1.357)	0.066
	Divorced	23(79.3%)	6(20.7%)	0.270(0.067, 1.093)	
	Widowed	16(69.6%)	7(30.4%)	3.77(0.188, 75.617)	
Educational status	Illiterate	29(34.1%)	56(65.9%)	1	
	Primary	74(48.7%)	78(51.3%)	0.992(0.364, 2.705)	0.007
	Secondary	78(71.6%)	31(28.4%)	0.413(0.144, 1.189)	
	College and above	40(25.9%)	14(25.9%)	0.183(0.053, 0.634)	
WHO stage	I	40(18.3%)	178(81.7%)	1	
	II	81(100%)	0(00%)	0.000	0.000
	III	81(98.8%)	1(1.2%)	0.001(0.000, 0.015)	
	IV	19(100%)	0(00%)	0.000	
OI prophylaxis given**	Yes	206(54.4%)	173(45.9%)	1	0.023
	No	15(71.4%)	6(28.6%)	0.223(0.061, 0.813)	
Baseline CD4 cell count, cells/mm^3^	<200	66(60.6%)	43(39.4%)	1	0.637
	$200	155(53.3%)	136(46.7%)	0.800(0.318, 2.017)	
Age (years)	18–29	100(56.8%)	76(43.21)	1	0.067
	30–39	63(57.3%)	47(42.7%)	1.175(0.478, 2.893)	
	40–49	43(53.8%)	37(46.3%)	1.387(0.521, 3.692)	
	$50	15(44.1%)	19(55.9%)	10.720(0.847, 135.678)	
Baseline hemoglobin level, g/dL	<10	21(44.7%)	26(55.3%)	1	0.226
	$10	200(56.7%)	153(43.3%)	1.857(0.682, 5.060)	
Baseline weight, kg	<60	72(48.3%)	77(51.7%)	1	0.521
	$60	149(59.4%)	102(40.6%)	0.757(0.324, 1.769)	

Note:**OI prophylaxis included Cotrimoxazole and/or INH

## Discussion

This study assessed the prevalence and associated factors of OIs among HIV-positive patients taking ART. The resulting study found that about 48.75% of HIV/AIDS patients on ART had one or more OIs. This finding was comparable to the 47.6% and 48% reported in a study conducted in Taiwan 12 and Eastern Ethiopia [14,21]. However, it is higher than two recent, similar studies carried out in Ethiopia in Debre Markos and Gondor, which reported 19.7% and 33.3% prevalence, respectively [16,22]. This difference might be due to the accessibility of higher health facilities and methodological differences in selecting study subjects. Similar studies were conducted among HIV patients taking ART for five and more years. The risk of developing an OI for a person receiving ART is highest during the initial month of therapy, shows that 39% after 12 months of initiation of ART [21]. There were 55 co-infections of different OIs observed in the current study.

Of these, 58.3% (n=44/55) were oral candidacies with ulcers - mouth, genital and (n=11/55) TB co-infections with herpes zoster and chronic diarrhea. This finding has disagreed with a report from Gondar, Ethiopia, which reported 50% TB and oral candidacies co-infections [22]. A higher proportion of oral candidacies with mouth ulcers, genital co-infections in the current study might be explained by a higher prevalence of these two OIs among the study participants. More than two OIs were also reported from studies conducted in Debre Markos, Ethiopia and Nigeria [16,23]. Oral candidacies were the most prevalent species isolated from both HIV seropositive subjects on ART 17 (76.19%) and that not on ART 8 (88.89%) [24]. The present study also revealed that oral candidacies infection is the predominant OI identified, with a prevalence of 11% (44/400). This was comparable with a study conducted in Abakaliki in which the prevalence of oral candidacies related OIs was found to be 12.5% [24], but, it was greater than the prevalence reported from East Ethiopia 7.5% and Nigeria (7.7%), which also reported TB as a major OI [23,25].

This might possibly be explained by methodological differences in selecting study subjects and the prevalence of oral candidacies in the total population. Next oral candidacies, herpes zoster, and TB were the second and the third most prevalent OIs in the present study, at 10.7% (43/400) and 9.5% (38/400), consequently. The prevalence of candidacies was higher compared with a report from Debre Markos, Ethiopia and a report from Nigeria in which prevalence rates of 11.8% and 8.6% were noted, respectively [16,23]. The prevalence of herpes zoster is in agreement with the study carried out in India, which revealed the prevalence of 14.7%. Eighteen, this rate was higher than the study reported in Nigeria [16] similarly, higher (30.7%) prevalence of HZ was reported in ART-naïve, HIV/AIDS-infected patients in Bahir Dar, Ethiopia [14]. This difference might be due to methodological differences in selecting study participants and the prevalence of HZ in the general population. The hospital where this study was conducted initiates ART when the CD4 level of a patient falls below 200 cells/mm^3^ of blood, which is far lower than the suggested by WHO, which increases the risk of HIV-infected individuals to OIs. In the current study, the HIV-infected patient with CD4 counts of 200 cells/mm^3^ was more similar to develop OIs compared to those with CD4 counts of $200 cells/mm^3^ [21,24-26].

## Conclusion

In this study, a high rate (55.3%) of OIs was reported. This suggests that OIs remain a challenge in patients receiving and taking ART in Ethiopia. Oral candidacies followed by herpes zoster and TB were the major OIs encountered by HIV-infected patients taking ART. Illiterate, WHO clinical stages III and IV were found to be strongly associated with the prevalence of OIs. That is why interventions need to be designed to promote early HIV testing and early enrollment of HIV-infected individuals into ART services. Individuals who continue to have advanced WHO clinical stage should be widely practiced in the routine management of people living with HIV, irrespective of ART use.

### What is known about this topic

- OIs were the cause of death in HIV infected individuals;
- WHO clinical stage develop when the immunity system of patients decreases and viral load increases;
- Proper prevention of mother-to-child transmission (PMTCT) service prevents HIV transmission from mother to child.

### What this study adds

- A high rate of OIs was reported. This suggests that OIs remain a challenge in patients receiving and taking ART;
- Oral candidacies followed by herpes zoster and TB were the major OIs encountered by HIV-infected patients taking ART;
- Individuals who continue to have advanced WHO clinical stage should be widely practiced in the routine management of people living with HIV, irrespective of ART use.
